# Trajectories of Screen Time across Adolescence and Their Associations with Adulthood Mental Health and Behavioral Outcomes

**DOI:** 10.1007/s10964-023-01782-x

**Published:** 2023-05-06

**Authors:** Xinxin Zhu, Helen Griffiths, Zhuoni Xiao, Denis Ribeaud, Manuel Eisner, Yi Yang, Aja Louise Murray

**Affiliations:** 1 Department of Psychology, University of Edinburgh, Edinburgh, UK; 2 Department of Clinical and Health Psychology, University of Edinburgh, Edinburgh, UK; 3 Jacobs Center for Productive Youth Development, University of Zurich, Zurich, Switzerland; 4 Institute of Criminology, University of Cambridge, Cambridge, UK

**Keywords:** Screen time, Mental health and behavioral issues, Adolescence, Joint trajectory

## Abstract

Excessive screen time among adolescents is discussed as a significant public health concern. Identifying adolescent longitudinal patterns of time spent on regularly-used media screens and understanding their young adulthood mental health and behavioral issue correlates may help inform strategies for improving these outcomes. This study aimed to characterize joint developmental patterns of time spent on videogames, surfing/chatting the Internet, and TV/DVDs during adolescence (at ages 11, 13, 15, 17) and their associations with mental health (i.e., depression, anxiety, suicidal ideation, and self-injury) and behavioral issues (i.e., substance use, delinquency, aggression) in early adulthood (at age 20). A parallel-process latent class growth analysis was used to model data from a diverse community-ascertained sample of youth in Zurich, Switzerland (*n* = 1521; 51.7% males). Results suggested that a five-class model best fitted the data: (1) low-screen use, 37.6%; (2) increasing chatting/surfing, 24.0%; (3) moderate-screen use, 18.6%; (4) early-adolescence screen use, 9.9%; and (5) increasing videogame and chatting/surfing, 9.9%. After adjusting for baseline levels of outcomes (primarily at age 11), the trajectory groups differed in their associations with adulthood outcomes of mental health and behavioral problems, indicating the importance of problematic screen usage patterns in predicting these outcomes. Future research to test the directionality of these associations will be important. These findings suggest which patterns of screen use may be a marker for later mental health and behavioral issues in different domains.

## Introduction

Adolescents’ excessive screen time has been found in some studies to be linked to negative well-being and development (e.g., physical, mental health, behavioral, and neurological problems) (see Fang et al., 2019; Neophytou et al., 2021, for reviews). However, these links are much debated, as it has also been suggested in other studies that, for example, the associations between adolescent screen time and mental health issues are inconsistent and small in magnitude, and that these associations have varied in terms of types of screen time as well as mental health problems (see, e.g., Tang et al., 2021, for a meta-analysis). These small effects or inconsistent findings may be due to the fact that a single assessment of youth screen time has typically been used in previous studies, whereas repeated measures throughout adolescence may provide a more accurate indication of longitudinal screen habits. Using developmental measures of screen time use can provide a more robust examination of their associations with well-being and development, as adolescents may change their screen time patterns (e.g., media types or activities) over time. It is critical to examine screen time patterns using a longitudinal framework. The current study aimed to evaluate the joint longitudinal patterns of time spent on three commonly used media types, i.e., TV/DVDs, videogames, and chatting/surfing on the Internet, over the entire adolescent period, as well as their associations with adulthood outcomes, including mental health (i.e., depression, anxiety, suicidal ideation, and self-injury) and behavioral (i.e., aggression, delinquency, and substance use) issues using data from a representative Swiss adolescent sample.

In adolescence, forming identities and forming/redefining relationships with peers (including romantic relationships) and parents are the “classic” developmental tasks. Digital media is an important place to fulfill these tasks for modern adolescents (Borca et al., 2015), and adolescence might be a time of changing needs with respect to screen media. Empirical research also supported this; for instance, adolescents’ time spent on media for social purposes (e.g., texting or chatting) increases during mid-adolescence and peaks in late adolescence, whereas their time spent on traditional media devices (e.g., television viewing, videogames) remains stable throughout adolescence (Coyne et al., 2018). Adolescence has been described as a time of increased arousal and vulnerability in emotional and behavioral regulation, which may pose a challenge for managing the escalating demands of specific media screens.

Empirical evidence has suggested the need to consider variations in adolescent developmental trajectories of screen time. For instance, one study estimated the developmental trajectories of time spent on TV in an Australian cohort (i.e., the Raine study, *n* = 2411) from childhood (5 years) to emerging adulthood (20 years) and identified three distinct patterns: consistently high, consistently low, and a sharp increase during the adolescent years (McVeigh et al., 2016). Considerable heterogeneity was also found in the developmental trajectories of time spent on texting in an American adolescent sample followed from ages 13 to 18 (*n* = 425), and four-trajectory groups were detected: perpetuals, decreasers, moderates, and increasers (Coyne et al., 2018). In addition, a study used the dataset of the Korean Youth Panel Study (KYPS, *n* = 3449) to evaluate the developmental patterns of adolescent students’ online game time, and a four-trajectory model was indicated: low, rising, declining, and chronic groups (Hong et al., 2014). Another study examined trajectories of total screen time (i.e., the sum of time spent on television, videogames, and computers) during a weekday in a Brazilian birth cohort followed from 11 to 18 years old (*n* = 3382) and found a three-group trajectory solution fitted the data best: always high, always moderate, and always low (Silva et al., 2017).

The aforementioned studies have provided evidence suggesting there is considerable heterogeneity in adolescent screen time developmental patterns. To date, most studies examining longitudinal trajectories of screen time in adolescence have evaluated a single media type (e.g., TV) or total screen time. Adolescents may have complex media use patterns, for example, spending a lot of time on several media types during a certain period of time or switching from one media type to another over successive periods of time. Thus, additional research is needed to characterize the longitudinal patterns of time spent on several frequently used media types among adolescents, which will allow us to build a more complete picture of their screen habits over time.

It may be clinically beneficial to identify young adults’ outcomes differentiated by the trajectory subgroups that emerge in the longitudinal pattern analyses (e.g., determined via latent class growth analysis, LCGA), as this could help illuminate the potential distress, costs, and impairment linked to particular trajectories. A small number of previous studies have also evaluated the subsequent correlates of identified screen time trajectory subgroups. For instance, studies have tested the associations of television watching trajectory classes across ages 5 to 20 with percentage body fat and mental health issues (including depression, anxiety, and stress) at age 20 (McVeigh et al., 2016), the associations between trajectories of texting from ages 13 to 18 and outcomes of depression, anxiety, aggression, relationship with parents and friends, and cell phone problems at age 18 (Coyne et al., 2018), and the associations between the trajectory subgroups of total screen time (including television, videogames, and computers) from 11 to 18 years and pulmonary function at 18 years (Silva et al., 2017). The findings of the abovementioned studies provide preliminary corroboration for the notion that adolescents who increasingly or persistently spend a lot of time on a particular type of media screen (or have greater total screen time) are more likely to develop poor outcomes. However, to the best of knowledge, no studies have examined the early adults’ correlates of distinct developmental trajectories jointly defined by multiple types of screen time. The parallel-process LCGA allows the developmental trajectories of several different constructs to be captured simultaneously, and the trajectory subgroups that emerge from this analysis can be used to compare subsequent outcomes. Such findings can potentially inform which screen use trajectories might be markers of mental health and behavioral issues in different domains and inform further research to examine causal directions of influence. For example, knowledge that a person might be a member of a problematic screen usage trajectory in adolescence might be used to inform preventive interventions to reduce the risk of adverse outcomes during early adulthood. A subset of outcomes that have previously been indicated to be related to at least one media type (i.e., TV/DVDs, videogames, and chatting/surfing on the Internet) were included in the present study to examine how screen-time trajectories associated with them. These outcomes include mental health and behavioral issues, i.e., depression, anxiety (e.g., Kandola et al., 2021; Stiglic & Viner, 2019), suicidal ideation (e.g., Coyne et al., 2021), self-injury (e.g., Wiguna et al., 2021), aggression (e.g., Keikha et al., 2020), substance use (e.g., Boers et al., 2020), and delinquency (Exelmans et al., 2015). These outcomes were included also as they are common in adolescence and tend to co-occur (e.g., Murray et al., 2022; World Health Organization, 2021).

Existing literature provides several theoretical frameworks that imply effects of screen time on these outcomes. Based on the displacement hypothesis (Kraut et al., 1998), all screen time can have a detrimental effect on health since it might replace time spent engaging in, for example, healthy pursuits (e.g., physical activity) or activities that might be beneficial to youth development (e.g., cognition, impulse control). Review work has suggested that prolonged sensory stimulation from excessive screen time could potentially exert adverse effects on brain development during adolescence, further associated with a wide range of impairments in cognitive function (e.g., deficits in reward and cognitive control), learning, and memory processes (see Marciano et al., 2021, for a review), which could in turn lead to issues in emotional and behavioral regulation. Another recent review study (Lissak, 2018) has proposed that the associations between excessive screen time and internalizing (e.g., depression and anxiety), externalizing (e.g., aggression), and suicidal behavior may be due to sleep problems, since excessive screen time may affect sleep in multiple ways (e.g., displacing other activities, after-dark use), and the association between sleep problems and mental health and behavioral issues is well-established.

Other theoretical perspectives have suggested that screen time effects may depend on the nature of the media content. For instance, chronic exposure to violent, suicidal-/self-injury-related, and substance-using content in videogames, TV/DVDs, and/or the Internet has been proposed to increase the risk of antisocial behavior (e.g., aggression, delinquency) (Bender et al., 2018), suicidality/self-injury (Arendt et al., 2019), and substance use (Davis et al., 2019), respectively. Besides this, media content (e.g., idealized body image) may drive youth to make upward social comparisons, likely associated with negative self-evaluation or emotional distress (e.g., Hanna et al., 2017). Taken together, building on the multiple theoretical perspectives that could explain associations between media use trajectories and mental health and behavioral outcomes, focusing on identifying trajectories that may be predictive of these issues may ultimately help inform the identification and/or prevention of them.

## Current Study

Given the paucity of research on the joint developmental trajectories of time spent on TV/DVDs, videogames, and chatting/surfing on the Internet throughout adolescence, the present study examined joint longitudinal patterns in these media types in a normative sample of adolescents measured at ages 11, 13, 15, and 17. This study also evaluated how the identified trajectory subgroups are associated with outcomes at age 20. As discussed above, in previous findings, three or more trajectories of media use (e.g., low, moderate, increasing, or high levels in a specific media screen) have typically been identified; this study hypothesized that at least three groups would be identified in the optimal model. These, the current study hypothesized, would include one group that spent little time on all three media types throughout adolescence, another group that spent an increasing amount of time chatting/surfing on the Internet as they approached late adolescence, and a third group that spent a moderate amount of time on one or more media types. Given the exploratory nature of the study, it was possible that more trajectories could emerge, which may provide further insights into common developmental patterns of media use and their implications. According to theoretical background (e.g., the displacement hypothesis and the potential detrimental effect of extended screen time on cognition function), prolonged exposure to media screens has been linked to mental and behavioral problems. The primary hypothesis of this study was that adolescents in a negative developmental pattern of screen time (e.g., spending increasing/large amounts of time on any media type) would have worse mental and behavioral outcomes at age 20 than those who spent little time on all three media types.

## Methods

### Participants

Data were drawn from an ongoing longitudinal study: the Zurich Project on Social Development from Childhood to Adulthood (z-proso, https://www.jacobscenter.uzh.ch/en/research/zproso/aboutus.html), with an initial target sample of *N* = 1675 urban youth. The sample is highly diverse in terms of the countries of origin and native languages of the parents (from around 80 different countries and about 50% non-native German speakers), and it is largely representative of Zurich’s youth population (Ribeaud et al., 2022). With regards to socioeconomic status, the mean International Socioeconomic Index (ISEI) (Ganzeboom, De Graaf, & Treiman, 1992) (based on data available at baseline) was 44.55 (*SD* = 17.75). This value approximately aligns with the occupational prestige level of a clerical worker. Table S1 of the supplementary materials provides a summary of the common birthplaces of caregivers. The first data collection was conducted in 2004/5 at the age of 7 and included 56 primary schools in the city of Zurich that were selected using a stratified random sample method that accounted for school size and socioeconomic background of school districts. This study used data from the fourth (age 11), fifth (age 13), sixth (age 15), seventh (age 17), and eighth (age 20) waves. Participants with valid data on variables of interest in at least one wave were included in the current analytic sample, and the analyses comprised a total of *n* = 1521 participants (51.7% males). In Switzerland, compulsory education lasts through the ninth grade (age 15/16 which corresponds to wave 6 in z-proso). The majority of youth (>90%) then pursue post-compulsory education, with about one-third pursuing baccalaureate level (up to age 18/19, grade 12), which leads to universities, and about two-thirds pursuing vocational training (which lasts for 2–4 years, equivalent to grades 11–13), which is typically a combination of school-based training in vocational schools and an apprenticeship in a company. At age 20 (wave 8 in z-proso), >50% of youth are still engaged in the education process. Previous papers provided additional information on the z-proso study and its non-response and attrition (e.g., Eisner et al., 2019; Ribeaud et al., 2022).

The z-proso study was approved by the responsible ethics committee at the Faculty of Arts and Social Sciences, University of Zurich, and participants’ parents or their own provided active informed consent at each wave included in the current study.

### Measures

#### Screen time

Screen time was assessed by six items, measuring the average time spent on media screens (TV/DVDs, videogames, and surfing/chatting on the Internet) on a normal school day and weekend day (e.g., “How many hours do you spend chatting and surfing on the Internet on a normal school day?”). Item responses were provided on a 5-point Likert scale (from *neve*r to *more than 3* *h/day*). The time spent on each type of media on weekdays and weekend days was separately assessed, which was combined to create a composite score. Previous studies have used comparable items to measure the time spent on these media types (e.g., Twenge & Campbell, 2018). The z-proso dataset grouped media screens as TV/DVDs, videogames, and chatting/internet surfing. TV/DVDs and videogames are typically viewed on a stationary device, whereas chatting and surfing the Internet are typically performed on a portable device. As portable devices can be used in more settings (e.g., when out and about) or at more times (e.g., before bed) than stationary devices, social media and internet use may have a greater effect on certain mental health and behavioral issues. Previous evidence has also suggested that time spent on social media and the Internet is more strongly associated with self-harm and depressive symptoms than time spent enjoying games or TV (Twenge & Farley, 2021). More research is required to determine if and how different media screens are associated with distinct outcomes.

#### Outcomes at age 20

Depression, anxiety, and aggression were assessed using the self-reported version of the Social Behavior Questionnaire (R. E. Tremblay et al., 1991). Its psychometric properties have been investigated by prior research utilizing the present sample (Murray, Eisner, et al., 2019; Murray, Obsuth, et al., 2019). There are eight items that measure depression and anxiety (each using four items) and nineteen items that measure aggression (e.g., physical, indirect, reactive, and proactive aggression). Responses were recorded using a five-point scale ranging from *“never” to “very often.”* Items were summed up to create composite scores for each dimension. Cronbach’s alphas for the depression, anxiety, and aggression subscales were 0.77, 0.79, and 0.85, respectively.

Suicidal ideation and self-injury were both measured by a single item that asked youth how often they had thought about suicide and intentionally self-injured during the past month, and they were rated on a five-point scale (1 = *never* to 5 = *very often*). These constructs were typically assessed by single items in previous studies (e.g., Donath et al., 2019; Perret et al., 2020).

Substance use (i.e., tobacco and cannabis use) was assessed by providing a checklist of substances along with the following instructions: “Listed below are some drugs, intoxicants and other substances. Have you ever taken any of them, and if yes, how many times in the *last 12 months*?” Tobacco and cannabis use were included in the current analysis, each substance was measured using one item on a six-point scale (from “*never”* to *“daily”*).

Delinquency was measured with a variety score that included responses to 7 items: stealing at home, shoplifting goods worth less than 50 CHF, shoplifting goods worth more than 50 CHF, vehicle theft, fare dodging, vandalism, and assault. The incidence of each behavior over a 12-month period was measured as a binary response, and the items were added together to create a composite score.

### Statistical Procedure

Parallel-process LCGAs were employed to examine joint longitudinal patterns of time spent on TV/DVDs, videogames, and chatting/surfing on the Internet. Both linear only models and linear+quadratic growth models were fitted to the data, and latent class models with one to eight class-solutions were evaluated both for exploratory purposes and for model parsimony. To determine the optimal model, the Lo-Mendall-Rubin (LMR) test was employed. This test determines whether a k-class trajectory model is significantly better than a k-1 class trajectory model, with a significant *p*-value indicating that the k-class model is statistically superior to the k-1 class model. The following criteria (as supplementary information) were used to determine the optimal model: Akaike information criterion (AIC), Bayesian information criterion (BIC), and sample size adjusted BIC (SaBIC), with lower values indicating better fit, and sufficient classification diagnostics (entropy, with values greater than 0.70 suggesting “acceptable” classification accuracy). To estimate the associations between the identified joint trajectory subgroups and adulthood outcomes, the three-step Bolck–Croon–Hagenaars procedures (BCH; Asparouhov, 2014) were used to add distal outcomes into the parallel-process LCGA. The BCH approach considers the probabilistic nature of class membership and produces more unbiased estimates than other methods (Bakk & Vermunt, 2016). The significance level was set at *α* = 0.05, and all tests were corrected for multiple comparisons using the Bonferroni correction. The Bonferroni correction has been criticized as being overly conservative, and both the raw *p* values and the Bonferroni-corrected threshold were reported based on the previous recommendation (VanderWeele & Mathur, 2019).

Pairwise comparisons of baseline-adjusted mental health and behavioral outcomes across trajectories were examined. Given that it is not possible to directly regress the outcomes onto the baseline levels of outcomes using the same model in the three-step BCH procedure, to achieve baseline adjustment, this study first regressed each outcome assessed at age 20 on the outcome first collected if it had not been collected at age 11 (depression, anxiety, aggression, and delinquency were collected at age 11, whereas self-injury was collected at age 13, and substance use and suicidal ideation were collected at age 15) in SPSS and saved their standardized residuals. These standard residuals represented outcomes adjusted for baseline levels of outcomes. Then, these standardized residual scores of the outcomes and their associations with trajectories of screen time were evaluated in the final LCGA models. Previous studies have suggested sex/gender differences in the association between screen time and mental health and behavioral issues (e.g., Tang et al., 2021), the results including sex as a covariate were also reported when estimating the associations between age 20 outcomes and screen time trajectories. This was implemented by regressing latent classes on sex.

Mplus 8.8 was employed to fit all models, which incorporated robust maximum likelihood (MLR) estimation. Missing data were addressed using the full information maximum likelihood (FIML) estimate, which produces unbiased parameter estimates when data are missing at random (MAR), as defined by Rubin (1976) (i.e., that the probability of missingness is independent of the missing values and dependent only on assessed variables included in the analysis).

## Results

### Descriptive Statistics

**Fig. 1:**
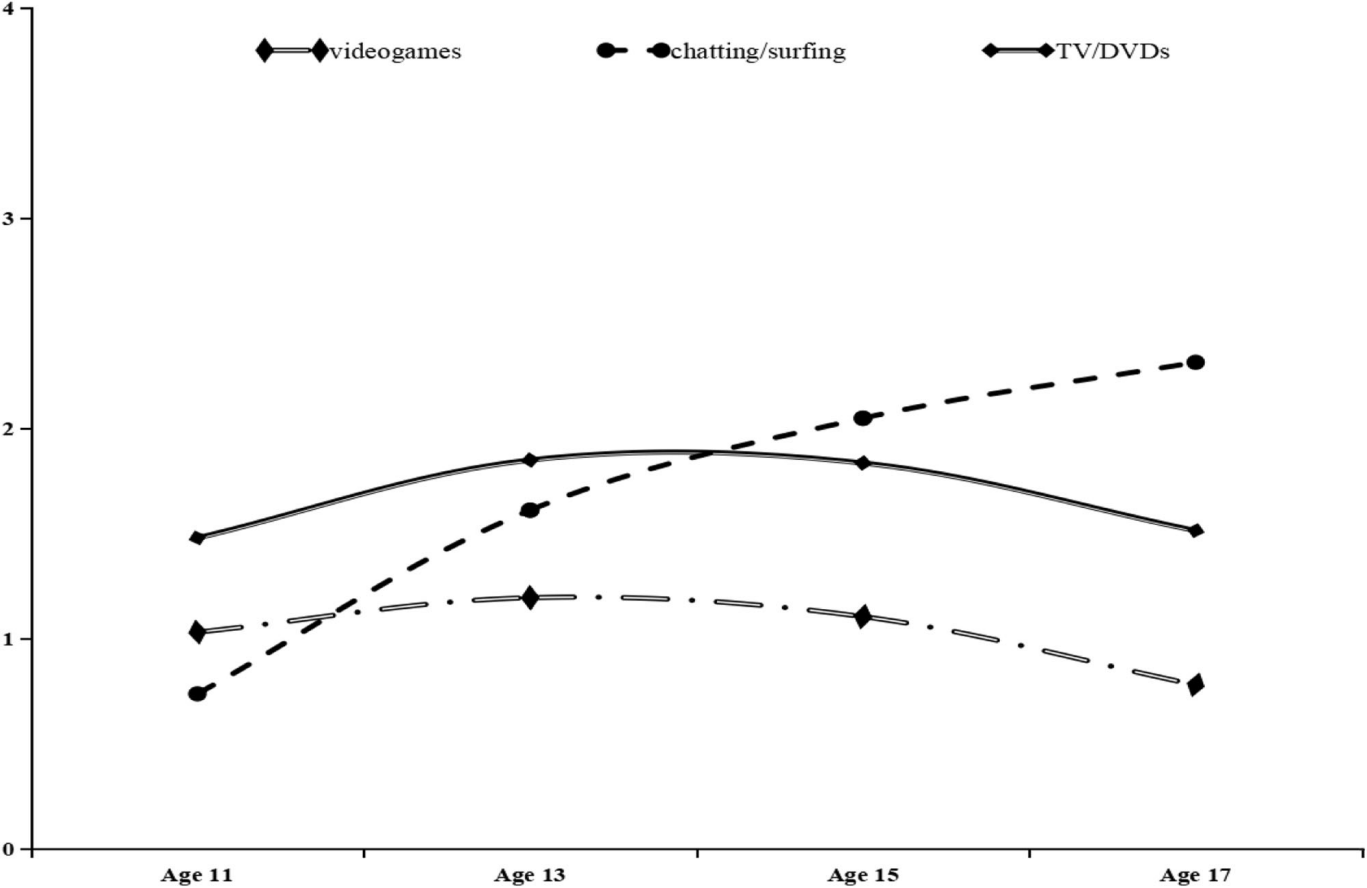
Average model-based trajectories of screen time on videogames, chatting/surfing, and TV/DVDs. The values on the vertical axis represent the number of hours per day, with 4 = more than 3 h per day

The descriptive statistics for the main study variables are provided in Table S2 in the supplementary materials. According to the values of AIC, BIC, and SaBIC, the results of model comparisons indicated that all linear+quadratic growth models fit the data better than their linear-only counterparts. The average model-based trajectory of multiple types of screen time was plotted based on the linear+quadratic growth model (see Fig. 1). The results indicated that time spent playing videogames and watching TV/DVDs remained relatively consistent throughout adolescence, whereas time spent on chatting/surfing the Internet increased.

### Parallel-Process LCGAs

**Fig. 2:**
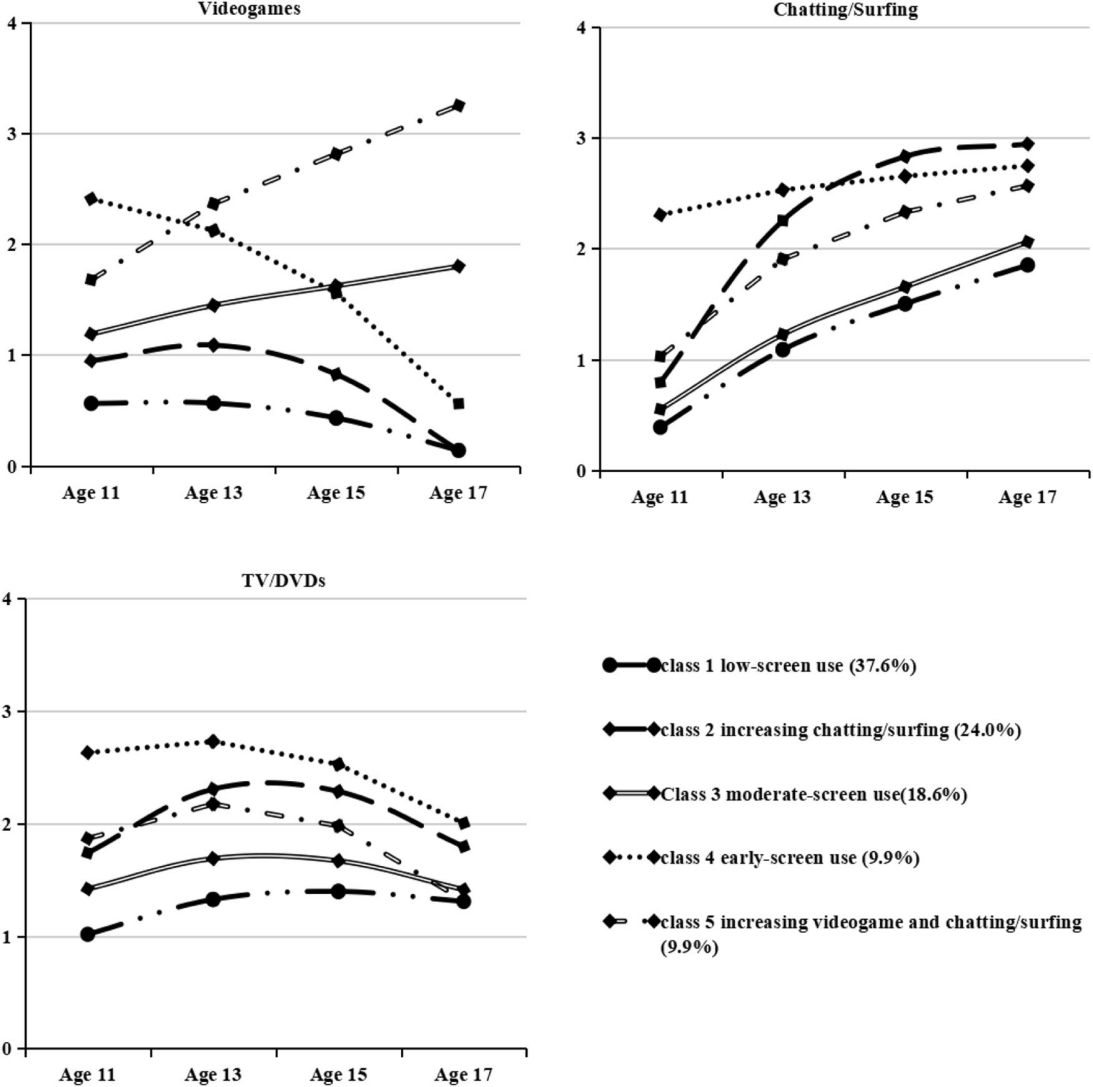
5-class model of screen time on videogames, chatting/surfing, and TV/DVDs. The values on the vertical axis represent the number of hours per day, with 4 = more than 3 h per day

The results of model fits, LMR test statistics, and entropy values for the 1–8 class models are summarized in Table 1. Entropy values for all models were close to or above 0.80, indicating good classification accuracy. The LMR test suggested that either a 3- or 5-class model could represent an optimal model, but the values of AIC, BIC, and SaBIC in the 5-class model were lower than those in the 3-class model; thus, the 5-class model was chosen as the final model. The model parameters for the 5-class model are shown in Table 2 and in Fig. 2. Participants in the first class (37.6% of the sample) initially spent about 0.5 h/day^1^ with a slight decrease and 1 h/day with a slight increase on videogames and TV/DVDs, respectively, whereas their time spent on chatting/surfing on the Internet was initially low (~0.5 h/day) and increased with age. This class was labeled “low-screen use.” Adolescents in the second class reported initially spending about 1 h/day on videogames but this decreased with age, initially spending about 1 h/day chatting/surfing on the Internet but this increased rapidly throughout adolescence, and initially spending about 2 h/day on TV/DVDs but this increased and then decreased with age. This class was labeled “increasing chatting/surfing.” Youth in the third class reported spending approximately 1 h/day and 0.5 h/day on videogames and chatting/surfing on the Internet, respectively. Both increased with age but only moderately. They initially spent approximately 1.5 h/day on TV/DVDs and this remained relatively stable throughout adolescence. This class was labeled “moderate-screen use.” The fourth group showed an initial, relatively high amount of time (~2.5 h/day) spent on videogames that decreased fast across adolescence. They also initially spent about 2.5 h/day on TV/DVDs, with this amount first increasing and then decreasing slightly throughout adolescence. They spent an initial relatively large amount of time (~2 h/day) on chatting/surfing on the Internet and this slightly increased with age. This group was labeled “early-adolescence screen use.” The fifth class reported initially spending about 2 h/day on videogames and rapidly escalating their use with age, initially spending about 1 h/day on chatting/surfing on the Internet, with this time increasing with age, and initially spending 2 h/day on TV/DVDs, with this time increasing then decreasing slightly throughout adolescence. This class was labeled “increasing videogame and chatting/surfing.”

**Table 1:** Model fits for the 1–8 class models

Model	LMR	*p*	AIC	BIC	saBIC	Entropy	Model	LMR	*p*	AIC	BIC	saBIC	Entropy
Model with linear and quadratic growth							Model with linear growth						
1-class	–	–	45857.858	45969.727	45903.016	N/A	1-class	–	–	46151.143	46247.032	46189.850	N/A
2-class	1709.690	<0.001	44144.834	44309.974	44211.495	0.754	2-class	1650.249	<0.001	44482.719	44615.897	44536.479	0.753
3-class	906.847	<0.001	43245.610	43464.023	43333.776	0.812	3-class	879.468	<0.001	43600.104	43770.572	43668.916	0.811
4-class	398.278	0.103	42861.896	43133.580	42971.566	0.823	4-class	375.685	0.098	43231.094	43438.852	43314.959	0.822
**5-class**	**348.580**	**0.018**	**42490.699**	**42815.653**	**42621.872**	**0.786**	5-class	301.768	0.014	42899.106	43144.154	42998.024	0.784
6-class	253.835	0.583	42253.399	42631.625	42406.077	0.795	6-class	238.210	0.067	42670.252	42952.590	42784.222	0.794
7-class	226.896	0.652	42043.407	42474.904	42217.588	0.805	7-class	212.882	0.115	42467.219	42786.847	42596.242	0.801
8-class	218.798	0.190	41841.622	42326.391	42037.307	0.804	8-class	124.032	0.471	486175.864	42711.687	42498.845	0.810

Solution(s) considered “best-fitting” indicated in bold

**Table 2:** Growth parameters for the selected 5-class model

Class Label (class size*)	Domain	Videogames			Surfing/chatting			TV/DVDs		
	Parameter	Intercept	Linear	Quadratic	Intercept	Linear	Quadratic	Intercept	Linear	Quadratic
Class 1 low-screen use (37.6%)	Estimate	0.56	0.27	−0.70	0.39	2.05	−0.59	1.02	1.13	−0.84
	SE	0.04	0.14	0.14	0.04	0.21	0.18	0.05	0.16	0.16
Class 2 increasing chatting/surfing (24.0%)	Estimate	0.95	1.10	−1.91	0.79	4.84	−2.69	1.74	2.36	−2.31
	SE	0.11	0.40	0.43	0.09	0.37	0.33	0.15	0.31	0.30
Class 3 moderate-screen use (18.6%)	Estimate	1.19	0.72	−0.11	0.55	1.92	−0.41	1.42	1.14	−1.15
	SE	0.07	0.31	0.32	0.06	0.25	0.24	0.09	0.27	0.25
Class 4 early-adolescence screen use (9.9%)	Estimate	2.41	−0.06	−1.79	2.30	0.67	−0.23	2.63	0.81	−1.44
	SE	0.19	0.84	0.77	0.28	1.02	0.78	0.11	0.52	0.44
Class 5 increasing videogame and chatting/surfing (9.9%)	Estimate	1.68	1.93	−0.35	1.03	2.76	−1.22	1.87	1.64	−2.19
	SE	0.13	0.47	0.42	0.12	0.52	0.48	0.15	0.48	0.43

*Based on estimated posterior probabilities

### Age 20 Outcomes of Joint Screen Time Trajectories

Group means and their comparisons for the five trajectory groups on each of the outcomes at age 20 are provided in Table 3. After adjusting for the baseline levels of the respective outcomes, the results showed that adolescents at age 20 in the class of increasing chatting/surfing reported significantly higher levels of depression compared to those in the subgroups of low-screen use and moderate-screen use, greater levels of aggression and tobacco use than those in the low-screen use class, and the highest levels of anxiety compared to those in the other four subgroups. Youth in the class of moderate-screen use showed greater aggression and tobacco use than those in the class of low-screen use, more delinquency than those in the classes of increasing chatting/surfing and early-adolescence screen use and low-screen use, and higher levels of cannabis use than those in the groups of increasing chatting/surfing and low-screen use, but were less likely to have anxiety than those in the class of low-screen use. Participants in the subgroup of early-adolescence screen use reported higher levels of aggression and tobacco use than those in the subgroups of low-screen use. Youth in the class of increasing videogame and chatting/surfing reported greater suicidal ideation than those in the class of low-and moderate-screen use, more aggression than those in the subgroup of low-screen use, higher levels of anxiety than moderate-screen use, and more self-injury than those in the subgroups of low-and moderate-screen use and increasing surfing/chatting. A number of key differences remained significant after applying the Bonferroni correction, specifically, for depression, increasing chatting/surfing >increasing videogame and chatting/surfing; for anxiety, increasing chatting/surfing >low-screen use & moderate-screen use & early-adolescence screen use, low-screen use >moderate-screen use; for aggression, increasing videogame and chatting/surfing & increasing chatting/surfing >low-screen use; for tobacco use, increasing chatting/surfing & early-adolescence screen use >low-screen use; for cannabis use and delinquency, moderate-screen use >low-screen use. Adjusting for the role of sex produced results that were generally comparable to those obtained without adjustment (see Table S3 in the supplementary materials).

**Table 3:** Comparison of age 20 outcomes by trajectory class based on parallel-process LCGA (Adjusting for baseline level)

Age 20 outcomes	Outcome means (SE) by class													
	low-screen use (c1)		increasing chatting/surfing (c2)			moderate-screen use (c3)			early-adolescence screen use (c4)				increasing videogame and chatting/surfing (c5)	
Outcomes’ mean at age 20														
Depression (range: 1–5)	2.36 (0.05)		2.60 (0.07)			2.38 (0.12)			2.23 (0.05)				2.42 (0.09)	
Anxiety (range: 1–5)	2.42 (0.05)		2.76 (0.08)			2.06 (0.06)			2.31 (0.12)				2.28 (0.09)	
Self-injury (range: 1–5)	1.12 (0.02)		1.10 (0.03)			1.07 (0.03)			1.19 (0.07)				1.29 (0.08)	
Suicidal ideation (range: 1–5)	1.27 (0.04)		1.34 (0.06)			1.23 (0.04)			1.27 (0.10)				1.53 (0.10)	
Aggression (range: 1–5)	1.33 (0.02)		1.49 (0.03)			1.43 (0.03)			1.58 (0.06)				1.55 (0.05)	
Tobacco use (range: 1–6)	3.22.(0.11)		4.11 (0.14)			3.66 (0.15)			4.60 (0.20)				3.62 (0.22)	
Cannabis use (range: 1–6)	2.40 (0.09)		2.34 (0.13)			2.92 (0.14)			2.95 (0.22)				2.57 (0.18)	
Delinquency (range: 0–7)	0.78 (0.05)		0.83 (0.07)			1.12 (0.07)			1.12 (0.14)				0.90 (0.11)	
Standardized residuals after adjusting for baseline levels of outcomes														
Depression	−0.03 (0.06)		0.24 (0.10)			−0.17 (0.07)			−0.09 (0.16)				−0.02 (0.12)	
Anxiety	0.02 (0.06)		0.38 (0.10)			−0.40 (0.07)			−0.18 (0.16)				0.00 (0.13)	
Self-injury	−0.01 (0.05)		−0.05 (0.07)			−0.10 (0.02)			0.04 (0.02)				0.35 (0.02)	
Suicidal ideation	−0.05 (0.05)		0.03 (0.09)			−0.07 (0.06)			−0.03 (0.15)				0.27 (0.14)	
Aggression	−0.24 (0.05)		0.22 (0.08)			0.03 (0.09)			0.18 (0.19)				0.31 (0.16)	
Tobacco use	−0.19 (0.06)		0.12 (0.07)			0.07 (0.08)			0.27 (0.12)				0.04 (0.12)	
Cannabis use	−0.10 (0.05)		−0.09 (0.08)			0.20 (0.08)			0.17 (0.13)				0.05 (0.12)	
Delinquency	−0.10 (0.05)		−0.04 (0.09)			0.27 (0.09)			−0.11 (0.17)				0.04 (0.15)	
Wald test *p* value	c1 vs. c2	c1 vs. c3		c1 vs. c4	c1 vs. c5		c2 vs. c3	c2 vs. c4		c2 vs. c5	c3 vs. c4	c3 vs. c5		c4 vs. c5
Depression	0.030*	0.139		0.735	0.893		0.001**	0.099		0.095	0.647	0.268		0.710
Anxiety	0.003**	<0.001***		0.241	0.885		<0.001***	0.005**		0.016*	0.222	0.008**		0.391
Self-injury	0.727	0.280		0.748	0.046*		0.580	0.635		0.037*	0.400	0.019*		0.187
Suicidal ideation	0.480	0.757		0.913	0.027*		0.331	0.736		0.139	0.797	0.025*		0.137
Aggression	<0.001***	0.013*		0.032*	0.001**		0.138	0.887		0.612	0.473	0.151		0.625
Tobacco use	0.002**	0.008**		<0.001***	0.084		0.621	0.317		0.563	0.161	0.843		0.181
Cannabis use	0.889	0.002**		0.060	0.229		0.011*	0.128		0.327	0.826	0.305		0.520
Delinquency	0.628	0.001**		0.933	0.384		0.016*	0.731		0.628	0.048*	0.227		0.500

Bonferroni adjusted α level = 0.005 (0.05/10). Bold values are statistically significant after Bonferroni’s correction. Pairwise comparisons (Wald test) were conducted.

## Discussion

Evaluation of the joint developmental trajectories of multiple types of media screen time throughout adolescence and their associations with young adulthood outcomes could provide a comprehensive understanding of adolescents’ longitudinal screen habits and early adults’ outcomes in order to inform strategies for improving these outcomes. This study used a parallel-process LCGA in a large longitudinal sample with 9 years of follow-up to estimate joint trajectory groups defined by the amount of time spent watching TV/DVDs, playing videogames, and chatting/surfing the Internet and their age 20 outcomes (including depression, anxiety, aggression, suicidal ideation, self-injury, substance use, and delinquency).

The findings suggested that, overall, from early to late adolescence, youth spent increasing amounts of time chatting/surfing on the Internet, but there was almost no increase in time spent on TV/DVDs and videogames, which is consistent with earlier research (Coyne et al., 2018). Previous findings also suggested adolescents use the Internet in the completion of their developmental tasks: e.g., exploring identity, developing and practicing autonomy, and formatting relationships outside the family (Borca et al., 2015). This may account for the increased time spent chatting/surfing on the Internet during this period. However, TV/DVDs and videogames are used for a variety of purposes (e.g., adolescents now increasingly use gaming platforms and online television viewing for social purposes) as technology advances during the longitudinal evaluation period of the current study, future studies would benefit from collecting the purpose of using media screens.

Moreover, the five trajectory subgroups found in this study indicated that the development of adolescents’ screen time was quite heterogeneous. The results showed that more than half of the participants (including low- and moderate-screen use) used each screen type for less than (or almost equal to) 2 h/day, yet their total screen time exceeded the recommended “less than 2 h/day” (M. S. Tremblay et al., 2016) for the majority of their adolescence. Given that these two groups spend the least time on screens compared to the other three groups, this means almost all adolescents, rather than a small group, may need to be encouraged to reduce the amount of time they spend on media screens, and this should be brought to the attention of parents and educators. Nearly a quarter of participants spent a rapidly increasing amount of time chatting/surfing online (from less than 1 h/day at age 11 to nearly 3 h/day at age 17). This group fits more closely with the overall trends previously discussed regarding the development of screen use in adolescence. Although screen use may link to some positive outcomes in terms of helping adolescents achieve developmental tasks, the concerns about the increasing use of screen time previously discussed suggest there is a need to better understand the pros and cons of different forms of screen use and the correlates of cumulative time spent on devices. About 10% of adolescents spent an increasing amount of time playing videogames (from ~1.5 h/day at age 11 to more than 3 h/day at age 17) and chatting/surfing online (from ~1 h/day at age 11 to ~2.5 h/day at age 17). The increased amount of time spent playing videogames in this group was inconsistent with the average trajectory observed for this type of screen, which more directly suggests that the heterogeneity of screen time trajectories should be taken into account. Additionally, ~10% of youth spent a lot of time playing videogames and watching TV/DVDs (~2.5 h/day for each type of screen) at age 11, and these youth may benefit from receiving guidance for screen use during their early adolescent years. Taken together, these findings indicate the complicated developmental patterns in adolescent screen use, and highlight the importance of monitoring changes in time spent on regularly used media screens in order to provide timely support for overuse behaviors (if their time on screen media exceeds the recommendation, i.e., 2 h/day). The heterogeneity of use also points to a need for tailoring programs based on screen-time trajectory group membership, as opposed to a “one size fits all” approach.

The current study also examined several adulthood outcomes associated with each screen time trajectory group. In terms of adulthood outcomes of depression and anxiety, the group of increasing chatting/surfing had the highest levels of these outcomes (though some comparisons were not statistically significant). These are consistent with some previous findings, for example, suggesting that spending more time on social media may lead to a particularly high risk of internalizing problems (Riehm et al., 2019). The aforementioned displacement hypothesis (e.g., displacing social interaction in the real world) and upward social comparisons offer two possible explanations for the association between chatting/surfing the Internet and internalizing problems. Some other speculative explanations include that excessive chatting/surfing online may increase dependence on social media/internet (see Lissak, 2018, for a review), that chatting/surfing online may increase the likelihood of negative social media experiences, such as cybervictimization (Craig et al., 2020), and that night-time use of screen media may lead to sleep disturbances (see Lissak, 2018, for a review), all of which may in turn lead to internalizing problems. On the other hand, this finding may be explained by the fact that youth at risk of internalizing problems are more likely to overuse the Internet. As one example, internalizing problems are associated with sleep difficulties, which may increase youth night-time use. Future studies are needed to investigate this possibility.

Regarding self-injury and suicidal ideation, youth in the trajectory of increasing videogame and chatting/surfing displayed a greater risk of these thoughts and behaviors at age 20. Previous research suggested that the acquired capability for suicide (including pain tolerance and fearlessness about death), i.e., a component of the interpersonal theory of suicide, may explain the link between videogames and suicidal behavior (e.g., Gauthier et al., 2014; Mitchell et al., 2015). This may be because overexposure to violent content in videogames and on the Internet may increase habituation to painful and/or frightening stimuli, which might increase the capability for suicide and then lead to self-injury or/and suicidality. This group also spent increased time chatting/surfing on the Internet during adolescence, as found in the increasing chatting/surfing group, which was associated with more internalizing symptoms; similarly, this group had relatively high levels of depressive symptoms, and the depression-suicide link has been well established in the existing literature. Since this group spent increased time both in videogames and chatting/surfing online during adolescence, additional research is necessary to determine whether the high levels of suicidality and self-injury exhibited by this group are due to their involvement in videogames or if there is a potential interaction between videogame use and chatting/surfing online. The aforementioned explanation was merely conjecture, and the association between time spent chatting/surfing on the Internet and videogames and suicidality/self-injury might be reciprocal. Existing research has paid much attention to the pathway from the former to the latter; further research would benefit from examining the directionality of this link.

Regarding aggressive behavior, adolescents in both the early-adolescence screen use group and the increasing videogame and chatting/surfing group exhibited higher levels of this behavior. A common explanation for this link pertains to the presence of violent media content within media activities (e.g., violent movie scenes on TV/DVDs, violent videogames, accessing violent images or videos through the Internet) (e.g., Holtz & Appel, 2011). According to the General Aggression Model (Anderson & Bushman, 2002), for example, exposure to violent content can lead to desensitization among individuals towards both real-life violence and media violence. This, in turn, may increase the probability of aggression due to repeated exposure to violent media and lead to the establishment of stable patterns of aggressive behavior. It should be noted that this explanation is just a speculation, and future research would benefit from delineating specific media content features (e.g., violent content, competition) that may contribute to such associations. Youth in the group of early-adolescence screen use also consumed more tobacco and cannabis at age 20 (the moderate-screen use group also showed greater cannabis use). As previously mentioned, review evidence has indicated that prolonged or/and repeated exposure to screen time during adolescence could lead to a reduction in the efficiency of the cognitive control system, as well as an inclination towards seeking short-term rewards (Marciano et al., 2021), which may be further associated with risk-taking behaviors such as substance use. In terms of delinquency, both the moderate-screen use and early-adolescence screen use groups had higher levels of it at age 20; however, after controlling for delinquency at age 11 and with Bonferroni correction, only the moderate-screen use group still had statistically significantly higher levels of delinquency compared to low-screen use, but not compared to other groups. This finding is potentially due to the fact that the current sample was sourced from the community and youth reported low levels of delinquency across groups. Again, it is worthwhile to mention the other possibilities regarding the association between screen time and behavioral problems (i.e., aggression, delinquency, and substance abuse). For example, adolescents with more behavioral problems may be more likely to be addicted to screens and pay more attention to violent content, possibly due to, e.g., their lower levels of self-control. This could have the potential to create a vicious circle and should be examined in future research. Besides this, adolescence may be a vulnerable period for developing these problems (i.e., aggression, delinquency, and substance use), since a developmental mismatch may exist where the socio-emotional system underlying sensation seeking matures more rapidly than the cognitive control system underlying self-regulation based on dual systems theories (Murray et al., 2021; Steinberg et al., 2008). Longer screen time, aggression, delinquency, and substance use may co-occur in adolescence. Additional research modeling their co-changes would be beneficial to illuminate their links across adolescent development.

Some interesting findings are worth noting, concerning the moderate-screen use group that showed significantly less anxiety but more cannabis use and delinquency than the low-screen use group. The potential explanation needs to be examined in future studies, but these findings might imply that moderately prolonged screen time is not always related to negative outcomes; it varies depending on the specific outcome in question. Future research needs to account for the possibility that there might be both positive and negative aspects to screen use (e.g., social connection versus exposure to cybervictimization, displacement, etc.). It should be noted that the above discussion regarding the potential mechanisms underlying the association between screen time trajectories and mental health and behavioral outcomes was speculative, and this association might be reciprocal (despite the fact that the current findings controlled for previous levels of outcomes), which should be investigated in future research.

### Implications of the Findings

The current study detected five trajectory subgroups, and they were associated with different risks for having negative mental health and behavioral issues in adulthood. These findings suggest the value of identifying the heterogeneity in adolescent longitudinal screen time and young adults’ outcomes and that aiming at screen time management to improve youth mental health and behavioral issues could be tailored to these sub-groups. It should be noted that the findings regarding the association between screen time trajectories and mental health and behavioral outcomes are correlational in nature, which provides limited information on the causality of these associations. The practical implications mentioned below would be more convincing if the causal directionality of these associations could be well established in future studies. The findings might suggest that screen time reduction intervention programs targeted at adolescents who spend an increasing amount of time on media screens, particularly chatting/surfing online and/or playing videogames, could help reduce the risk of developing mental health problems including depression, anxiety, suicidal ideation, and self-injury. Interventions aimed at youth who spend much time playing videogames in early adolescence could aid in reducing behavioral issues (e.g., aggression and substance use). However, as mentioned earlier, future research will be required to rule out reverse causality given that youth at risk of mental health issues may be more drawn to spending higher and/or increasing amounts of time engaged in screen use. Existing literature suggests several strategies that may be used to reduce screen time in adolescents, such as self-monitoring, behavioral contracts, screen time budgeting, and parental newsletters focusing on reducing screen time (e.g., Lubans et al., 2016; Salmon et al., 2011). Regardless of the direction of causality, the current findings point to the fact that screen use histories may flag the risk of a range of mental health and behavioral issues. Those displaying high or escalating levels may thus benefit from screening for mental health or behavioral issues.

### Limitations and Future Directions

The limitations of this study should be noted. First, the assessment of screen time was based on self-reported measures, and the categorization of media variables was limited by the measures available. Future studies using objective (accelerometer-based) measures and with detailed categorization (e.g., separately assessing chatting and surfing the Internet) will provide a more accurate record of screen time and a more precise examination of the link between time spent on specific media activities and mental health and behavioral issues. Second, some important characteristics (e.g., suicide-related content) and purposes (e.g., learning or entertainment) of media screen activities were not assessed in the z-proso study and these characteristics could help to understand the outcomes associated with each type of screen time. Third, the current study only focused on a unidirectional pathway from screen time to mental health and behavioral issues, however, there might be a bidirectional association between these constructs (e.g., Yang et al., 2022). Fourth, future studies employing designs, e.g., the experience sampling method, to repeatedly measure screen time in briefer intervals or to passively record media use will provide more detailed information on adolescent usage patterns and their association with consequences, whilst overcoming possible recall biases associated with traditional questionnaire methods. Fifth, gender/sex differences in the trajectory of screen time and its association with mental health and behavioral problems are not the focus of current research, future studies should examine possible sex/gender moderating effects using large sample sizes. Sixth, data collection for this study began about 14 years ago, during which time there have been significant changes in media consumption/use patterns, and new media activities need to be evaluated to contribute to the understanding of more recent youth screen usage and its association with mental health and behavioral problems. Finally, the current study was limited by focusing only on negative outcomes and youth time spent on screen media. Future research should evaluate positive outcomes (e.g., subjective well-being) and collect additional information such as youth time spent on other activities (e.g., physical activities) and the availability of other activities that might moderate the link between screen time and mental health and behavioral issues.

## Conclusion

There is a lack of studies using a longitudinal framework to evaluate joint trajectories of time spent on several regularly-used media screens throughout adolescence and their associations with adulthood mental and behavioral outcomes. This could help provide a more comprehensive understanding of the development of adolescent screen habits and help improve young adults’ these problems. To address this knowledge gap, this study used parallel-process LCGA to summarize the joint trajectories of time spent on TV/DVDs, videogames, and chatting/surfing the Internet during adolescence and their relations with adulthood mental health and behavioral problems. Findings may suggest that promoting healthy media device habits among youth who spend an increasing amount of time chatting/surfing the Internet and/or playing videogames in adolescence, and youth who spend a great deal of time playing videogames in early adolescence could help improve their mental health (i.e., depression, anxiety, suicidality, self-injury) and behavioral (i.e., aggression and tobacco and cannabis use) problems, respectively. These patterns can serve as markers of mental health/behavioral issue risk and can be used to identify young people who may benefit from being screened for these issues. However, the current findings provide only initial evidence regarding intervention implications. To provide stronger evidence, more research is required to evaluate the bidirectional associations between screen time and mental health and behavioral issues, and to understand youth screen media use in a broader developmental context, such as the engagement in a variety of activities (e.g., physical activities) in their developmental context.

## Supplementary Information

Electronic Supplementary Materials

## Footnotes

1 This daily screen time (including all references to screen time per day below) is calculated by averaging the screen time used on weekdays and weekends.
