# Supplementary material for: Trajectories of Screen Time across Adolescence and Their Associations with Adulthood Mental Health and Behavioral Outcomes
Source: J Youth Adolesc. 2023 May 6;52(7):1433–47. doi: 10.1007/s10964-023-01782-x (PMC10175337; doi:10.1007/s10964-023-01782-x)
Supplement: Supplementary file 1 — Electronic Supplementary Materials [file 10964_2023_1782_MOESM1_ESM.docx]

**Table S1: Common primary caregivers’ birth countries (n=1521)**

| **Nationalities** | **Number** | **Percentage** |
| --- | --- | --- |
| Switzerland | 744 | 48.9% |
| Serbia-Montenegro | 103 | 6.8% |
| Portugal | 88 | 5.8% |
| Sri Lanka | 82 | 5.4% |
| Germany | 57 | 3.7% |
| Italy | 54 | 3.6% |
| Turkey | 52 | 3.4% |

**Table S2: Descriptive statistics**

| Constructs | *n* | *M* | *SD* | Minimum | Maximum |
| --- | --- | --- | --- | --- | --- |
| Age 11 Videogames | 1129 | 1.02 | 0.89 | 1 | 5 |
| Age 13 Videogames | 1353 | 1.24 | 1.13 | 1 | 5 |
| Age 15 Videogames | 1440 | 1.05 | 1.15 | 1 | 5 |
| Age 17 Videogames | 1293 | 0.80 | 1.11 | 1 | 5 |
| Age 11 Chatting/surfing | 1131 | 0.73 | 0.85 | 1 | 5 |
| Age 13 Chatting/surfing | 1349 | 1.65 | 1.09 | 1 | 5 |
| Age 15 Chatting/surfing | 1440 | 2.00 | 1.17 | 1 | 5 |
| Age 17 Chatting/surfing | 1291 | 2.33 | 1.12 | 1 | 5 |
| Age 11 TV/DVDs | 1131 | 1.48 | 0.92 | 1 | 5 |
| Age 13 TV/DVDs | 1350 | 1.83 | 1.02 | 1 | 5 |
| Age 15 TV/DVDs | 1441 | 1.85 | 1.04 | 1 | 5 |
| Age 17 TV/DVDs | 1290 | 1.51 | 1.04 | 1 | 5 |
| Age 20 Depression | 1180 | 2.40 | 0.85 | 1 | 5 |
| Age 20 Anxiety | 1180 | 2.40 | 0.92 | 1 | 5 |
| Age 20 Self-injury | 1180 | 1.13 | 0.48 | 1 | 5 |
| Age 20 Suicidal ideation | 1178 | 1.30 | 0.71 | 1 | 5 |
| Age 20 Aggression | 1180 | 1.43 | 0.36 | 1 | 3.78 |
| Age 20 Tobacco use | 1177 | 3.68 | 1.92 | 1 | 6 |
| Age 20 Cannabis use | 1178 | 2.55 | 1.67 | 1 | 6 |
| Age 20 Delinquency | 1179 | 0.90 | 0.95 | 0 | 6 |

**Table S3: Comparison of age 20 outcomes by trajectory class based on parallel-process LCGA controlling for sex (Adjusting for baseline level)**

|  | Outcome means (SE) by class | | | | | | | | | | | | | |
| --- | --- | --- | --- | --- | --- | --- | --- | --- | --- | --- | --- | --- | --- | --- |
| **Age 20 outcomes** | low-screen use (c1) | | increasing chatting/surfing (c2) | | | moderate-screen use(c3) | | | early-adolescence screen use (c4) | | | | increasing videogame and chatting/surfing (c5) | |
| **Outcomes’ mean at age 20** |  | |  | | |  | | |  | | | |  | |
| Depression (range: 1-5) | 2.36 (.05) | | 2.60 (.07) | | | 2.23 (.05) | | | 2.37 (.12) | | | | 2.42 (.08) | |
| Anxiety (range: 1-5) | 2.42 (.05) | | 2.78 (.08) | | | 2.05 (.06) | | | 2.28 (.12) | | | | 2.28 (.09) | |
| Self-injury (range: 1-5) | 1.12 (.02) | | 1.08 (.03) | | | 1.08 (.03) | | | 1.19 (.07) | | | | 1.27 (.07) | |
| Suicidal ideation (range: 1-5) | 1.26 (.04) | | 1.32 (.06) | | | 1.25 (.05) | | | 1.26 (.10) | | | | 1.51 (.09) | |
| Aggression (range: 1-5) | 1.34 (.02) | | 1.48 (.03) | | | 1.43 (.03) | | | 1.57 (.05) | | | | 1.56 (.05) | |
| Tobacco use (range: 1-6) | 3.24 (.11) | | 4.04 (.13) | | | 3.67 (.15) | | | 4.72 (.20) | | | | 3.64 (.20) | |
| Cannabis use (range: 1-6) | 2.42 (.09) | | 2.27 (.12) | | | 2.88 (.14) | | | 3.13 (.23) | | | | 2.59 (.17) | |
| Delinquency (range: 0-7) | .81 (.05) | | .79 (.07) | | | 1.09 (.07) | | | 1.17 (.14) | | | | .94 (.11) | |
| **Standardized residuals after adjusting for baseline levels of outcomes** | | | | | | | | | | | | | | |
| Depression | -0.03 (.06) | | 0.23 (.09) | | | -0.16 (.07) | | | -0.06 (.17) | | | | -0.02 (.11) | |
| Anxiety | 0.01 (.06) | | 0.36 (.09) | | | -0.38 (.07) | | | -0.13 (.17) | | | | -0.02 (.12) | |
| Self-injury | -0.01 (.05) | | -0.09 (.06) | | | -0.06 (.07) | | | 0.03 (.15) | | | | 0.30 (.16) | |
| Suicidal ideation | -0.05 (.05) | | 0.00 (.08) | | | -0.03 (.06) | | | -0.01 (.14) | | | | 0.25 (.13) | |
| Aggression | -0.23 (.05) | | 0.16 (.08) | | | 0.03 (.09) | | | 0.21 (.19) | | | | 0.37 (.16) | |
| Tobacco use | -0.18 (.06) | | 0.12 (.07) | | | 0.05 (.08) | | | 0.29 (.12) | | | | 0.05 (.11) | |
| Cannabis use | -0.09 (.05) | | -0.10 (.07) | | | 0.16 (.08) | | | 0.24 (.14) | | | | 0.07 (.11) | |
| Delinquency | -0.08 (.05) | | -0.10 (.08) | | | 0.24 (.09) | | | -0.04 (.17) | | | | 0.09 (.14) | |
| **Wald test *p* value** | c1 vs. c2 | c1 vs. c3 | | c1 vs. c4 | c1 vs. c5 | | c2 vs. c3 | c2 vs. c4 | | c2 vs. c5 | c3 vs. c4 | c3 vs. c5 | | c4 vs. c5 |
| Depression | .031* | .184 | | .868 | .905 | | **.001**** | .148 | | .081 | .593 | .293 | | .828 |
| Anxiety | **.004**** | **<.001***** | | .429 | .807 | | **<.001***** | .016* | | .011* | .171 | .013* | | .611 |
| Self-injury | .382 | .559 | | .787 | .065 | | .812 | .477 | | .025* | .565 | .051 | | .228 |
| Suicidal ideation | .587 | .736 | | .790 | .026* | | .781 | .921 | | .108 | .941 | .067 | | .182 |
| Aggression | **<.001***** | .020* | | .021* | **<.001***** | | .274 | .819 | | .246 | .386 | .081 | | .522 |
| Tobacco use | **.002**** | .019* | | **<.001***** | .061 | | .498 | .264 | | .608 | .105 | .984 | | .164 |
| Cannabis use | .973 | .013* | | .023* | .181 | | .023* | .043* | | .214 | .606 | .533 | | .332 |
| Delinquency | .829 | **.005**** | | .817 | .268 | | .008** | .749 | | .248 | .171 | .417 | | .578 |

*Note: Bonferroni adjusted α level=.005 (.05/10). Bold values are statistically significant after Bonferroni’s correction. Pairwise comparisons (Wald test) were conducted.*
